# Supplementary material for: Antibiotic Use: A Cross-Sectional Study Evaluating the Understanding, Usage and Perspectives of Medical Students and Pathfinders of a Public Defence University in Malaysia
Source: Antibiotics (Basel). 2019 Sep 19;8(3):154. doi: 10.3390/antibiotics8030154 (PMC6784178; doi:10.3390/antibiotics8030154)
Supplement: Supplementary file 1 [file antibiotics-08-00154-s001.pdf]

Appendix A

Questionnaire Regarding Antibiotic Use: A Cross-Sectional Study Evaluating the Understanding, Usage and Perspectives of Medical Students and Pathfinders of a Public Defence University in Malaysia

First Part: Demographic Data

1–Year of birth

1a–Age

2–Gender: M F

3–Place of birth (Country)____

4–Place of birth of the father (Country)______________________________

5–Place of birth of the mother (Country)______________________________

6–Does at least one member of your family (parents, children, husband/wife) work in a health-related field?

□ yes

□ no

7–Year of Study: 1/2/3/4/5

8–Session:

9–Ethnic Group:

10–Marital Status: Married/Unmarried/Divorce

11–Grade of Obtained in either Last Professional/Semester:

12–Are you a Cadet Officer/Territorial Army/Civil Student?

Second Part: Frequency of Antibiotic Use

7–Have you used antibiotics in the last year?

□ yes

□ no

8–If yes, how many times?

□ 1–2

□ 3–5

□ >5

Third Part: Knowledge about Antibiotics

9–Penicillin or Amoxicillin are antibiotics.

1	2	3	4
Total disagreement			Total agreement

10–Aspirin is an antibiotic.

1	2	3	4
Total disagreement			Total agreement

11–Paracetamol is an antibiotic.

1	2	3	4
Total disagreement			Total agreement

12–Antibiotics are useful for bacterial infections (e.g., Tuberculosis).

1	2	3	4
Total disagreement			Total agreement

13–Antibiotics are useful for viral infections (e.g., flu).

1	2	3	4
Total disagreement			Total agreement

14–Antibiotics are indicated to reduce any kind of pain and inflammation.

1	2	3	4
Total disagreement			Total agreement

15–Antibiotics can kill "good bacteria" present in our organism.

1	2	3	4
Total disagreement			Total agreement

16–Antibiotics can cause secondary infections after killing good bacteria present in our organism

1	2	3	4
Total disagreement			Total agreement

17–Antibiotics can cause allergic reactions.

1	2	3	4
Total disagreement			Total agreement

Fourth Part: Awareness about Antibiotic Resistance

18–Have you ever heard about antibiotic resistance?

- ☐ yes
- ☐ no

19–In particular, have you discussed the problem of antibiotic resistance during degree courses?

- ☐ yes
- ☐ no

20–Have you ever heard of it outside degree courses? If yes, where have you heard it from? (More than one answer is possible)

- ☐ I have never heard about it outside a degree course
- ☐ General Practitioner
- ☐ Television
- ☐ Newspaper
- ☐ Web
- ☐ Other____________________

21–Antibiotic resistance is a phenomenon for which a bacterium loses its sensitivity to an antibiotic.

1	2	3	4
Total disagreement			Total agreement

22–Misuse of antibiotics can lead to a loss of sensitivity of an antibiotic to a specific pathogen.

1	2	3	4
Total disagreement			Total agreement

23–If symptoms improve before it is completed the full course of antibiotic, you can stop taking it.

1	2	3	4
Total disagreement			Total agreement

Fifth Part: Attitudes Regarding Consumption of Antibiotics

24–Do you usually take antibiotics for the cold or a sore throat?

- ☐ yes
- ☐ no

25–Do you usually take antibiotics for fever?

- ☐ yes
- ☐ no

26–Do you usually stop taking antibiotics when you start feeling better?

- ☐ yes
- ☐ no

27–Do you take antibiotic only when prescribed by the doctor?

- ☐ yes
- ☐ no

28–Do you keep leftovers antibiotics at home because they might be useful in the future?

- ☐ yes
- ☐ no

29–Do you use leftovers antibiotics when you have cold, sore throat or flu without consulting your doctor?

- ☐ yes
- ☐ no

30–Do you buy antibiotics without a medical receipt?

- ☐ yes
- ☐ no

31–Have you ever started an antibiotic therapy after a simple doctor call, without a proper medical examination?

- ☐ yes
- ☐ no
